# Impact of the Extracellular Vesicles Derived From *Trypanosoma cruzi*: A Paradox in Host Response and Lipid Metabolism Modulation

**DOI:** 10.3389/fcimb.2021.768124

**Published:** 2021-10-28

**Authors:** Heloisa D’Avila, Núbia Pereira de Souza, Ana Luíza da Silva Albertoni, Laíris Cunha Campos, Pollianne Garbero Rampinelli, José Raimundo Correa, Patrícia Elaine de Almeida

**Affiliations:** ^1^ Laboratory of Cellular Biology, Department of Biology, Federal University of Juiz de Fora (UFJF), Minas Gerais, Brazil; ^2^ Laboratory of Microscopy and Microanalysis, Department of Cell Biology, University of Brasilia, Brasilia, Brazil

**Keywords:** extracellular vesicles, *T. cruzi*, infectious diseases, inflammation, lipid droplets, prostaglandin, parasite replication, Changas disease

## Abstract

Chagas disease is a major public health problem, especially in the South and Central America region. Its incidence is related to poverty and presents a high rate of morbidity and mortality. The pathogenesis of Chagas disease is complex and involves many interactive pathways between the hosts and the *Trypanosoma cruzi*. Several factors have been implicated in parasite-host interactions, including molecules secreted by infected cells, lipid mediators and most recent, extracellular vesicles (EVs). The EVs of *T*. *cruzi* (EVsT) were reported for the first time in the epimastigote forms about 42 years ago. The EVsT are involved in paracrine communication during the infection and can have an important role in the inflammatory modulation and parasite escape mechanism. However, the mechanisms by which EVs employ their pathological effects are not yet understood. The EVsT seem to participate in the activation of macrophages *via* TLR2 triggering the production of cytokines and a range of other molecules, thus modulating the host immune response which promotes the parasite survival. Moreover, new insights have demonstrated that EVsT induce lipid body formation and PGE_2_ synthesis in macrophages. This phenomenon is followed by the inhibition of the synthesis of pro-inflammatory cytokines and antigen presentation, causing decreased parasitic molecules and allowing intracellular parasite survival. Therefore, this mini review aims to discuss the role of the EVs from *T. cruzi* as well as its involvement in the mechanisms that regulate the host immune response in the lipid metabolism and its significance for the Chagas disease pathophysiology.

## Introduction

Chagas disease is a neglected disease caused by infection with *Trypanosoma cruzi*, in which the persistence of the parasite and the prolonged activation of the immune system lead to a chronic inflammatory process and cardiomyopathies (D’Avila et al., 2011). The pathogenesis of Chagas disease is a multifactorial complex mechanism that involves a large number of molecules and vesicles, among them, the extracellular vesicles (EVs) – small lipid vesicles released from the host- cell and/or parasite-cell into the extracellular space, potentially modulating the immune response (Cestari et al., 2012; Torró et al., 2018).

The EVs traffic has been the target of important mechanisms of cellular communication. Moreover, the EVs can mediate parasite-parasite and host-parasite interactions. Infected cell-derived EVs induce the communication between distant parasites and facilitate the dissemination of virulence factors (Mantel and Marti, 2014). In addition, EVs from parasitic protozoa are important in the pathogenicity and disease progression (Gonçalves et al., 1991; Nogueira et al., 2015).

The persistence of parasitemia through immune evasion mechanisms demonstrates the success of *T. cruzi* in the chronic development of Chagas disease where the EVs play an important role in modulating the immune response to the parasite. The purpose of this mini review is to present the recent progress in elucidating the origin, morphology and functions of EVs from the host cells and *T. cruzi*, as well as their impact on the parasite escape mechanism.

## Extracellular Vesicles Origin and Morphology

The term extracellular vesicles (EVs) is commonly used to indicate different membrane-bound structures delimited by a lipid bilayer, released in the extracellular environment. The EVs are heterogeneous, with different biogenesis, molecular composition, sizes (from 20 nm to 5 µm) and functions (Van Niel et al., 2018; Witwer and Théry, 2019). They are secreted by either prokaryotic or eukaryotic cells, thus extending their phenotype (Campos et al., 2010; Torrecilhas et al., 2012). Moreover, EVs can carry and transfer molecules for the maintenance of homeostasis, respond to cellular imbalance and help the rapid modulation and/or evasion of the immune response during different pathogenic infections (Oliveira et al., 2010; Marcilla et al., 2014; Vargas et al., 2015; De Pablos et al., 2016; Dong et al., 2019; Babatunde and Subramanian, 2020; Palacios et al., 2021).

The EVs populations are usually classified according to their origin and size into three different types: exosomes (20–100 nm), microvesicles (MVs) (ectosomes like EVs - 100–1,000 nm) and apoptotic bodies (>1000 nm) (Akers et al., 2013; Witwer and Théry, 2019). The main type of EVs are categorized according to their intracellular origin in eukaryotic cells: the exosomes, formed inside multivesicular bodies and released upon fusion of these endosomal compartments with the plasma membrane and the microparticles, formed by direct budding and constriction from the plasma membrane (Akers et al., 2013). In EVs from trypanosomatids, the vesicles have been described as larger vesicles that bud from the plasma membrane and smaller vesicles that bud within the flagellar pocket. They are released through exocytosis of multivesicular bodies carrying components of the parasite membrane and intracellular environment (Silveira et al., 1979; Lovo-Martins et al., 2018). In general, EVs from parasites or hosts cells are isolated from the culture after spontaneous secretion, then the supernatants containing EVs are filtered for a total exosome isolation (Cestari et al., 2012; Lovo-Martins et al., 2018) (**Table 1**).

**Table 1:** Extracellular vesicles (EVs) in *T. cruzi* and other parasites.

Pathogen	Reference	EV	Origin	Study model	Metodology
*T. cruzi*	Bautista-López et al., 2017	M / E	Trypomastigotes (Tulahuen strain) / Vero cells	*in vitro*	UC, proteomic, WB, SEM
	Bayer-Santos et al., 2013	M / E	Epimastigotes and trypomastigotes (Dm28c clone)	*in vitro*	UC, proteomic, NTA,Sucrose-density gradient, TEM
	Cestari et al., 2012	M	THP-1 and mouse blood (BALB/c mice)	*in vitro* and *in vivo*	UC, FC, TEM
	Choudhuri and Garg, 2020	M	Trypomastigotes (SylvioX10/4, ATCC 50823) / C2C12, Raw 264.7, blood samples (WT and Parp1^-/-^)	*in vitro* and *in vivo*	UC, NTA, ZetaView, PCR, CL-ELISA, WB
	Cronemberger-Andrade et al., 2020	M / E	Trypomastigotes (Y strain) / THP-1	*in vitro*	UC, SEC, proteomic, SEM, NTA
	De Pablos et al., 2016	M / E	Trypomastigotes (CL- Brener, PAN4 strains)	*in vivo*	UC, TEM
	Garcia-Silva et al., 2014	M / E	Trypomastigotes (Dm 28c clone)	*in vitro*	UC, TEM, Bradford
	Lovo-Martins et al., 2018	M / E	Trypomastigotes (Y strain)	*in vitro* and *in vivo*	UC, NTA
	Lozano et al., 2017	M / E	Amastigote, epimastigotes, trypomastigotes (PAN4 strain) / Vero cells	*in vivo*	UC, SEM, TEM, confocal laser scanning microscopy, Micro-BCA, WB
	Madeira et al., 2021	M / E	Trypomastigotes (Y strain) and Plasma of chronic Chagas disease patients	*in vitro* and *in vivo*	UC, CL-ELISA, NTA, SEM
	Moreira et al., 2019	E	Trypomastigotes (PAN4 strain)	*in vitro*	UC, TEM, NTA, DLS, WB, Micro-BCA
	Neves et al., 2014	M / E	Trypomastigotes (Y strain and CL-Brener clone)	*in vitro* and *in vivo*	UC, SEM, TEM, Bradford
	Nogueira et al., 2015	M	Trypomastigotes (Colombiana, YuYu, Y and CL-14 strain)	*in vitro* and *in vivo*	UC, SEC, SEM, NTA, CL-ELISA, Micro BCA
	Ramirez et al., 2017	M	Epimastigotes and trypomastigotes (Sylvio X10/6, Y, CL strains ) / THP‐1	*in vitro* and *in vivo*	UC, FC, proteomic, Bradford
	Ribeiro et al., 2018	M / E	Trypomastigotes (Y and YuYu strains)	*in vitro*	UC, SEC, SEM, NTA, CL-ELISA, WB, proteomic
Others					
*Toxoplasma gondii*	Beauvillain et al., 2007	E	Dendritic cell (SRDC)	*in vivo*	UC, SEM, Micro-BCA
*Leishmania amazonensis*	Cronemberger-Andrade et al., 2014	M / E	Macrophages (Bone marrow cells - BALB/c mice )	*in vitro*	UC, FC, TEM, Micro-BCA
*Mycobacterium bovis BCG* and *M. tuberculosis*	Giri and Schorey, 2008	E	*M. tuberculosis* and *M. bovis* BCG-infected macrophages (J774 cell line)	*in vitro* and *in vivo*	UC, Sucrose-density gradient, FC, Micro-BCA, WB, TEM
*Leishmania infantum, L. braziliensis* and *L. amazonensis*	Nogueira et al., 2020	M / E	*L. infantum* (MCAN/BR/89/BA262 strain), *L. braziliensis* (MHOM/BR/01/BA788 strain), and *L. amazonensis* (MHOM/BR/87/BA125 strain) promastigotes	*in vitro* and *in vivo*	UC, NTA, SEM, Micro-BCA
*Cryptococcus neoformans*	Oliveira et al., 2010	M / E	*C. neoformans* (HEC3393, B3501, Cap 67 strains)	*in vitro*	UC, confocal laser scanning microscopy, quantitative fluorimetric Amplex Red sterol assay kit
*Leishmania donovani* and *L. major*	Silverman et al., 2010	E	*L. donovani* (Sudan S2, 1SR, 1SR HSP1002/2, Bob, BobLPG22/2 strains) and *L. major* (Fredlin strain) amastigotes	*in vitro* and *in vivo*	UC, mass spectrometry, Sucrose-density gradient, WB
*Candida albicans*	Vargas et al., 2015	M / E	*C. albicans (*11, ATCC 90028, ATCC SC5314 strains)	*in vitro*	UC, TEM, DLS, SDS-PAGE, immunoblotting, proteomic, quantitative fluorimetric kit

M, Microvesicles; E, Exosomes; UC, Ultracentrifugation; WB, Western Blot; SEM, Scanning Electron Microscopy; TEM, Transmission Electron Microscopy; FC, Flow Cytometry; DLS, Dynamic Light Scattering; NTA, Nanoparticle Tracking Analysis; SEC, Size-exclusion Chromatography; CL-ELISA, Chemiluminescent Enzyme-linked Immunosorbent Assay; Micro-BCA, bicinchoninic acid assay; Micro-BCA, protein assay kit; PCR, Polymerase Chain Reaction; CLSM, Confocal Laser Scanning Microscopy.

The EVs participate in intercellular communication (parasite-parasite, parasite-host cell or host cell-host cell), modulate the immune response and act as pro-inflammatory mediators (Lovo-Martins et al., 2018). Thus, they can potentiate the course of the infection from the delivery or capture of its content by the host cells, through distinct routes: endocrine, paracrine, juxtacrine or autocrine signaling (Torró et al., 2018). These pathways, however, can be influenced by several factors such as the phase of the disease, immunocompromised patient, levels of parasitemia and parasite life cycle (Ramirez et al., 2017; Torró et al., 2018).

## Role of EVs From *T. cruzi* (EVsT) in the Imunne System Modulation

For years, numerous studies have been conducted on EVs in the context of diseases (Silveira et al., 1979; Lovo-Martins et al., 2018; Cronemberger-Andrade et al., 2020). The EVs are constantly shedding and sharing their products with the extra- or intracellular milieu which is correlated with the niche for proliferation and survival used by the different pathogens. The role and impact of EVs secreted by parasites during infection have been highlighted in several studies. However, the capacity of EVs to modulate the host-cell response is not clear.

**Figure 1:**
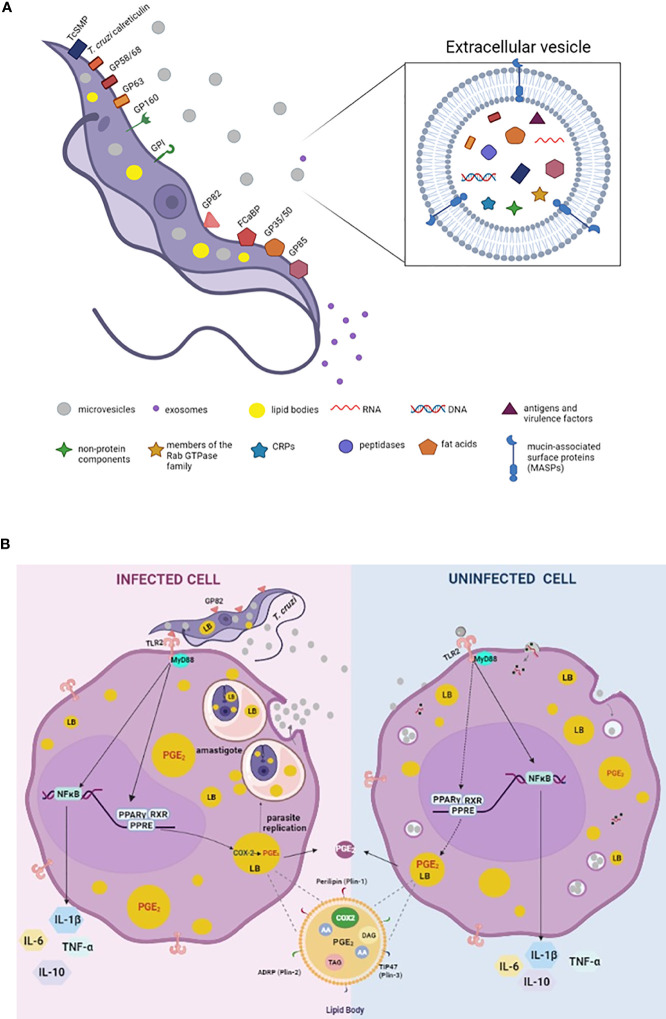
Schematic model summarizing the molecules involved on parasite-host cell interaction process and EVs secreted by trypomastigotes of *Trypanosoma cruzi* **(A)**. Mechanisms of action triggered by *T. cruzi*, EVs and EVsT in macrophages **(B)**. **(A)** Parasite exhibiting major components of its membrane associated with EVsT. Note the parasite cytoplasm containing microvesicles, exosomes, and lipid bodies (LBs). In detail, EVsT showing a lipid bilayer containing the main macromolecules carried by these structures, such as DNA, RNA, fatty acids, enzymes, mucin-associated surface proteins (MASP), among others. **(B)** *T. cruzi* internalized by macrophage (infected cell) induces LB formation and PGE_2_ derived from LBs contributing to amastigote replication, as well as release of microvesicles from the host cell (EVs). We suggest that the EVsT from *T. cruzi* and EVs from infected host cells are also recognized by uninfected macrophages *via* TLR2 inducing LB formation and PGE_2_ synthesis (dashed lines). However, further studies are needed to define the receptors and signaling responses induced upon EVs and EVsT-macrophage interaction and how these interactions/responses change as the exosome composition is modified during an infection. In general, *T cruzi* and/or EVs/EVsT *via* TLR2 active PPAR-y to translocate to nucleus, heterodimerize with RXR and binding to specific DNA response elements (PPRE) in target genes, altering the lipid metabolism and inducing LB formation. The PGE_2_ (produced in LB_S_ *via* COX-2 enzyme activation) is a potent lipid mediator which reduces the host Th1 immune response (by inhibition of the TNF-α and IL-6 and induction of the IL-10) and down modulates the microbicidal function of the macrophage. In parallel, the activation of NK-κB also modulates the synthesis of cytokines. In detail, representation of the structure and composition of LB. Arachidonic acid (AA); cyclooxygenase -2 (COX-2), triacylglycerol (TAG), diacylglycerol (DAG), peroxisome proliferator-activated receptor-y response element (PPER).

The EVsT are produced in the different life cycle phases of the parasite and participate in the host cell infection process (Torrecilhas et al., 2020). In addition, several signaling cascades are activated by EVsT components modulating host-cell responses (Torrecilhas et al., 2020) (**Figure 1A**). They can modulate the cytoskeleton as well as the invasion of the metacyclic trypomastigotes by inducing tyrosine kinase phosphorylation and the actin nucleation (Yoshida and Cortez, 2008; Torrecilhas et al., 2020).

In *T. cruzi* infection, it is proposed that the intracellular life cycle should be responsible not only for parasite-parasite transmission but also for parasite-host cells, since the EVsT develop in a compartmentalized environment which increases their probability of coupling and delivery within the host cell (Torró et al., 2018). Thus, the EVsT can function as effectors in the host-parasite interaction mechanisms modulating the host immune response, increasing the number of cells infected as well as the parasitaemia (Moreira et al., 2019).

The role of the EVs in the immune system modulation is controversial. In general, some studies have demonstrated that the EVs present a protective role (Oliveira et al., 2010; Cronemberger-Andrade et al., 2014; Nogueira et al., 2015; Ribeiro et al., 2018). However, in other studies, the EVsT have a role in the evasion mechanism of the immune response, followed by an increase (50% to 250%) in parasitaemia in mice cells (Lovo-Martins et al., 2018; Moreira et al., 2019).

In the early stages of *T. cruzi* infection, the parasites promote the release of vesicles from the host cell plasma membrane, which may contribute to their survival (Cestari et al., 2012; Bautista-López et al., 2017). According to Ramirez et al., 2017, the close contact between the membranes results in the bidirectional fusion of microvesicles (host-parasite and vice-versa), thus facilitating the interaction between the parasite and the host cell plasma membranes. In metacyclic, tissue culture-derived trypomastigote and noninfective epimastigote, *T. cruzi* forms were shown to induce different levels of EVs release from host cells (Ramirez et al., 2017). Furthermore, the EVs released during the interaction of the parasite with host cells were able to increase (around 40-50%) the host cell invasion by metacyclic trypomastigotes (Ramirez et al., 2017).

In the *T. cruzi* infection, the mobilization of intracellular Ca^2+^ deposits by the host cell during the cell invasion leads to the depolarization of the host plasma membrane, the depolymerization of F-actin and to lysosomal recruitment to the point of infection (Tardieux et al., 1994; Caler et al., 2000; Scharfstein et al., 2000). The family of small membrane proteins of *T. cruzi* (TcSMP) detected in the EVsT trigger Ca^2+^ signaling and mobilization/exocytosis of lysosomes, events that induces parasitophorous vacuoles formation and invasion of the parasite (Neves et al., 2014; Martins et al., 2015). Thus, contributing to the invasion of host cells and the increase in the percentage of cellular parasitism (Moreira et al., 2019).

Furthermore, the evasion of the complement-mediated response is triggered by the formation of EVs in host cells that are induced by metacyclic trypomastigotes (Cestari et al., 2012; Lidani et al., 2017). The release of EVs from the parasitized cells is done as an escape mechanism of the innate immunity response, by invading host cells and inhibiting complement-mediated lysis and also facilitating host cell invasion. The parasite has been shown to be able to escape the immune system by depositing host cell-derived EVs on its surface, which inhibits the action of C3 convertase (Cestari et al., 2012). Once trypomastigotes reach the bloodstream, the parasite bypasses complement-mediated lysis and opsonization with the aid of surface proteins such as calreticulin and the complement regulatory protein (CRP) also called GP160. The Gp160 and the conserved regions of the N- and C-terminal of the mucin-associated surface proteins (MASPs) were found in the EVs secreted by trypomastigotes (De Pablos et al., 2016). The Gp160 is a trypomastigote GPI anchor surface protein that binds to C3b and C4b dissociating the classical and alternative C3 convertase from complement (Norris et al., 1991; Norris, 1998).

The EVs can also contribute to the activation of the immune response with the release of proinflammatory cytokines (Torró et al., 2018). Different strains of *T. cruzi* have been shown to release EVsT and promote the activation of macrophages *via* TLR2 (Nogueira et al., 2015). Cronemberger-Andrade et al., 2020, showed that THP-1 cells infected by EVsT were able to induce the activation and translocation of NF-κB *via* TLR2 signaling. In addition, the EVs released by uninfected THP-1 cells also activated the cells *via* TLR2 (Cronemberger-Andrade et al., 2020). Furthermore, the EVs enriched with α-galactosyl triggered proinflammatory responses in macrophages *via* TLR2-signaling pathway (Nogueira et al., 2015; Ribeiro et al., 2018), (**Figure 1B**). Also, Choudhuri and Garg, 2020, demonstrated a proinflammatory response in macrophages stimulated by EVsT and EVs from infected cells and plasma of acutely and chronically infected mice, in a mechanism dependent on PARP1 (a DNA repair enzyme). EVs containing oxidized DNA fragments are recognized by cytosolic DNA sensors, cyclic GMP-AMP synthase (cGAS) and as consequence they synergize with PARP1 inducing a NF-κB-mediated proinflammatory cytokine production (Choudhuri and Garg, 2020).

However, in infection-derived inflammatory processes, the pre-treatment with EVsT from Y strain (EVsY) can downmodulate the release of TNF-α and nitric oxide (around 50%) as well as the increase in cardiac parasitism (2.5 times) in mice (Lovo-Martins et al., 2018). These data were associated with a reduction in TNF-α in plasma, decreased production of TNF-α and IL-6 by the spleen cells of infected mice (Lovo-Martins et al., 2018). Also, macrophage stimulation with EVsY before infection by *T. cruzi* increased (around 100%) the internalization rate of the parasite and the release of infecting trypomastigotes by these cells (Lovo-Martins et al., 2018), (**Figures 1A, B**).

Recently, Madeira et al., 2021, observed a lower concentration of circulating EVs associated with differential activation of the immunological system in patients with chronic Chagas disease, with increased production of IFN-*γ*, when compared with uninfected healthy controls (Madeira et al., 2021). This data was associated with parasite persistence suggesting that the EVs can be potential candidates as biomarkers during the course of Chagas disease.

## Composition of EVsT And Its impact In The Course of Chagas Disease

The EVs composition contains proteins involved in host-parasite interactions, signaling, traffic and membrane fusion, transporters, oxidation-reduction, oxidized DNA, small RNAs derived from tRNAs and rRNAs among others (Théry et al., 2002; Bayer-Santos et al., 2013; Bautista-López et al., 2017; Witwer and Théry, 2019; Choudhuri and Garg, 2020), (**Figure 1A**). Moreover, EVs contain specific proteins involved in vesicle formation and specific markers of the endosomal pathway, such as Rab GTPases, chaperones and tetraspanins (Ostrowski et al., 2010).

The EVsT also carry a wide range of potential virulence factors, such as peptidases (calpain cysteine peptidase, oligopeptidase, thermostable carboxypeptidase 1 or aminopeptidase P), responsible for the proteolysis of different peptide substrates (Alvarez et al., 2012), oxidized DNA (Choudhuri and Garg, 2020) and ribosomal subunit (Torró et al., 2018). The epimastigotes forms also release fragments of tRNA that can induce epigenetic changes in host cells, changing the expression profile of genes involved with cytoskeleton, extracellular matrix and immune response pathways (Garcia-Silva et al., 2014; Torrecilhas et al., 2020).

The probability of the EVsT to reach distant cells is related to the cellular microenvironment of the infected tissue and its distance to the appropriate means of transport (blood and lymphatic fluid) (Rank et al., 2011; Torró et al., 2018). In addition, different post-translational modifications in the EVs nucleus shared by the different forms of the parasite can also cause changes in the vesicle composition, protein targets and/or biological functions (Torró et al., 2018). Bayer-Santos et al., 2013, analyzed the proteomic composition of EVsT, confirming that a large proportion of the *T. cruzi* secretome is constitutively released *via* EVs (Bayer-Santos et al., 2013).

It has been demonstrated that *T. cruzi* PAMPs are poorly detected by the innate immune system at the beginning of the disease, delaying the activation of the immune response of infected host cells (Torró et al., 2018). By immuno electron microscopic analysis, on the surface of the EVsT it was found mucin-associated proteins (MSPs), that induced an insufficient switching from IgM to IgG during the infection in mice, allowing the parasite to escape the humoral response (De Pablos et al., 2016). Similar data was observed in chagasic patients (Lozano et al., 2017).

In general, these studies suggest that EVsT are able to modulate the inflammatory response by inhibition of pro-inflammatory cytokine and NO production, as well as alteration on the humoral response in favor of the parasite (Torrecilhas et al., 2009; De Pablos et al., 2016; Lozano et al., 2017; Lovo-Martins et al., 2018).

In summary, the studies show that there is no consensus on the role of EVs during the acute and chronic phases of Chagas disease. Most of the studies discussed here during the acute phase of the disease suggest that EVs and EVsT collaborate with the development of the parasite escape mechanism. In the chronic phase there is insufficient IgG exchange during infection in mice, which allows the parasite to escape the humoral response, Choudhuri and Garg, 2020, demonstrating that EVs contained damaged DNA, thus collaborating to a pro-inflammatory profile during the chronic phase. (Choudhuri and Garg, 2020).

## *T. cruzi* and EVsT Elicits Lipid Body Biogenesis and Lipid Mediator Synthesis in Macrophages During Infection

Lipid body (LB) accumulation within macrophages is a common feature observed in models of Chagas disease and other parasite infections (D’Avila et al., 2008; Almeida et al., 2018). In *in vitro* and *in vivo T. cruzi* infection, LBs are present in both, host and parasite cells (Toledo et al., 2016). Moreover, EVsT induce LB formation and lipid mediator synthesis that modulate the host response in favor of the parasite (Lovo-Martins et al., 2018).

The mechanism of biogenesis of LBs is a regulated event highlighting the role of peroxisome proliferator-activated receptor-γ (PPAR-γ), a member of the nuclear receptor family. When activated, PPAR-γ acts as a transcription factor, translocating to the nucleus and heterodimers with the retinoid X receptors in target genes. (**Figure 1B**) The PPAR-γ is involved in the mechanisms of synthesis of inflammatory mediators, fatty acid uptake and lipid storage in macrophages. Also, the activation of the TLR2 initiates a signaling cascade that culminates in LB biogenesis through the PPAR-γ activation (Almeida et al., 2009; Almeida et al., 2014). This data suggests that the TLR2 activation by EVsT may also activate PPAR-γ translocation during the mechanism of LB formation in Chagas disease (**Figure 1B**).

In unstimulated cells, LBs are present in small numbers. However, in cells involved in inflammatory processes, infectious or non-infectious, the number of LBs can increase considerably, depending on the type of cell and the stimulus (Murphy, 2012). In the experimental infection by *T. cruzi* in rats and mice, it was demonstrated that the parasite promotes an intense inflammatory response characterized by monocytes migration to the infectious sites (Freire-De-Lima et al., 2000; Melo et al., 2003). In these sites, LBs formation in macrophages was associated with enhancement of parasitism, characterized by increased numbers of parasite nests in cardiomyocytes (Melo et al., 2003; Toledo et al., 2016).

Although not presenting a typical membrane, LBs are delimited by an electron-dense hemi-membrane formed by a hydrophilic monolayer composed of phospholipids and structural proteins. The hydrophobic core consists mainly of neutral lipids such as triacylglycerol (TAG – the major components of LBs), diacylglycerol (DAG) and cholesterol ester, as well as unsaturated fatty acids, such as arachidonic acid (AA) and oleic acid (OA) (Murphy, 2001; Walther et al., 2017).

The LBs hemi-membrane display structural proteins from perilipin family (PLIN), including perilipin/PLIN1, PLIN2/ADRP (for “adipose differentiation-related protein”), PLIN3/TIP47 (for “tail-interacting protein of 47 Kda”) (**Figure 1B**). The LBs present proteins involved in cell signaling processes, in vesicular transport, histones and cytokines in eukaryotic cells (Bozza et al., 2009; Li et al., 2012). Therefore, the LBs directly or indirectly act as hubs for many cell functions, such as metabolic processes, energy, store of neutral lipids for membrane synthesis, membrane traffic, intracellular signaling, lipid metabolism and the production of several inflammatory mediators (Walther et al., 2017; Almeida et al., 2018).

These organelles are considered intracellular sites of substrates and enzymes involved in the synthesis of lipid mediators biologically active, such as eicosanoids (D’Avila et al., 2006; D’Avila et al., 2011; Almeida et al., 2018). Arachidonic acid (AA) is the precursor of the eicosanoids, which is metabolized by enzymes, such as cyclooxygenase-2 (COX-2), to produce lipid mediators such as prostaglandins (PGs). Earlier studies have demonstrated that during *T. cruzi* infection, macrophages were positively immunostained for COX-2, (Freire-De-Lima et al., 2000; D’Avila et al., 2011). Although a COX-like enzyme has been reported in parasites, they do not express mammalian homologues COX-1 or COX-2. Thus, a synthesis of PGs is performed by PG synthases, which has already been identified in parasites with homology to humans (Kubata et al., 2007). Trypomastigotes stimulated with AA showed an increased number of LBs, representing sites of PGE_2_ synthase (Toledo et al., 2016). In all these studies, both macrophages and parasite released large amounts of PGE_2_ from new formed-LBs.

In summary, these data support the hypothesis that PGE_2_ synthesis derived from LBs in infected cells are involved in the production of inflammatory mediators which can potentially inhibit the Th1 response in the host promoting the replication and survival of the parasite *via* enhancement of IL-10 production and a drastic reduction of TNF-α (D’Avila et al., 2011; Kalinski, 2012). Moreover, *T. cruzi* LBs are involved in the mechanism of release of the immunosuppressive inflammatory mediators, acting as an evasion strategy by the parasite (Toledo et al., 2016).

Interestingly, LBs biogenesis and PGE_2_ synthesis have been observed in infected cells and in cells that do not contain internalized parasites, suggesting paracrine stimulation or a bystander amplification for the formation of these organelles and PGE_2_ derived from LBs during *in vivo* and *in vitro* infections (Melo et al., 2003; D’ Avila et al., 2011). This intercellular communication can occur through host-host cells or host-parasite mediators. Corroborating this fact, Lovo-Martins et al., 2018, demonstrated that EVsT from the Y strain alone were able to induce LBs and PGE_2_ production by macrophages. In addition to LB formation, EVsT-stimulated macrophages showed higher PGE_2_ production than non-stimulated macrophages (Lovo-Martins et al., 2018). PGE_2_ derived from LBs inhibit the synthesis of TNF-α and antigen presentation, causing decreased NO production, thus allowing intracellular parasite survival (Freire-De-Lima et al., 2000; D’ Avila et al., 2011).

Lovo-Martins et al., 2018, also demonstrated that infected macrophages primed with EVsT produced more PGE_2_ (10 times) and less TNF-α and IL-6 (around 90% and 80%, respectively) than infected macrophages without prior EVsT exposure (Lovo-Martins et al., 2018). These authors hypothesized that EVsT could be down modulating the expression and activity of COX-2. As a result, the immune modulation exerted by PGE_2_ production induced by EVsT seems to be important specifically in the beginning of the infection. In general, these data suggest that EVsT create a more favourable environment for *T. cruzi* infection, with a reduction in inflammatory cytokines and in the trypanocidal molecule NO.

These data support the role of EVsT in the complex pathogenesis of the acute phase of Chagas disease and provide new insights for a better understanding of the parasite-host interaction. However, the functionality of the EVs and the charges they carry in their compartments as well as the relevance of these products to the host cell should be further studied, as little is known about the ability of the EVs to modulate the conditions of the host cell.

## Conclusion

The EVs shedding is a highly conserved parasite-host-cell interaction mechanism. The interaction between *T. cruzi* and its host cells is a bidirectional phenomenon, with thousands of EVs shared during the process. In this mini-review, we discuss the paradoxical role of EVs, which might coexist and affect differently the host response, presenting on one hand a protective role while in other studies, it contributes to the evasion mechanism, mainly through the modulation of the lipid metabolism for the production of PGE_2_. Also, in this mini-review, we mentioned several typical implications of EVs during *T. cruzi* infection, with an important impact in the host-lipid metabolism even in uninfected cells. Thus, the importance of EVs in the modulation of the host immune response presents a potential target for biomarkers of the Chagas disease progression.

## Author Contributions

HD and PA drafted the manuscript. AA and LC edited figures. HD, AA, NS, LC, PR, JC, and PA wrote and approved the final version of the paper. PA and HD edited the manuscript. All authors contributed to the article and approved the submitted version.

## Funding

This work was supported by grants from Fundação de Amparo à Pesquisa de Minas Gerais (FAPEMIG), Conselho Nacional de Desenvolvimento Científico e Tecnológico do Brasil (CNPq) (309523/2019-2), FAPDF and Programa de Pós-Graduação em Patologia Molecular/UNB. AA is PhD student supported by a UFJF (Federal University of Juiz de Fora) fellowship, NS and LC are PhD students supported by CAPES (Coordenação de Aperfeiçoamento de Pessoal de Nível Superior) fellowship.

## Conflict of Interest

The authors declare that the research was conducted in the absence of any commercial or financial relationships that could be construed as a potential conflict of interest.

## Publisher’s Note

All claims expressed in this article are solely those of the authors and do not necessarily represent those of their affiliated organizations, or those of the publisher, the editors and the reviewers. Any product that may be evaluated in this article, or claim that may be made by its manufacturer, is not guaranteed or endorsed by the publisher.

## References

[B1] Akers J. C. Gonda D. Kim R. Carter B. S. Chen C. C. (2013). Biogenesis of Extracellular Vesicles (EV): Exosomes, Microvesicles, Retrovirus-Like Vesicles, and Apoptotic Bodies. J. Neurooncol. 1 (113), 1–115. doi: 10.1007/s11060-013-1084-8 PMC5533094 23456661

[B2] Almeida P. E. Roque N. R. Magalhães K. G. Mattos K. A. Teixeira L. Maya-Monteiro C.. (2014). Differential TLR2 Downstream Signaling Regulates Lipid Metabolism and Cytokine Production Triggered by *Mycobacterium bovis BCG* Infection. Biochim. Biophys. Acta - Mol. Cell Biol. Lipids 1841 (1), 97–107. doi: 10.1016/j.bbalip.2013.10.008 24120921

[B3] Almeida P. E. Silva A. R. Maya-Monteiro C. M. Torocsik D. D’Avila H. Dezso B.. (2009). *Mycobacterium bovis bacillus calmette-Guerin* Infection Induces TLR2-Dependent Peroxisome Proliferator-Activated Receptor Expression and Activation: Functions in Inflammation, Lipid Metabolism, and Pathogenesis. J. Immunol. 183 (2), 1337–1345. doi: 10.1515/crll.1891.108.269 19561094

[B4] Almeida P. E. de Toledo D. A. M. Rodrigues G. S. C. D’Avila H. (2018). Lipid Bodies as Sites of Prostaglandin E2 Synthesis During Chagas Disease: Impact in the Parasite Escape Mechanism. Front. Microbiol. 9, 499 (Mar). doi: 10.3389/fmicb.2018.00499 29616011 PMC5869919

[B5] Alvarez V. E. Niemirowicz G. T. Cazzulo J. J. (2012). The Peptidases of *Trypanosoma Cruzi*: Digestive Enzymes, Virulence Factors, and Mediators of Autophagy and Programmed Cell Death. Biochim. Biophys. Acta - Proteins Proteomics 1824 (1), 195–206. doi: 10.1016/j.bbapap.2011.05.011 21621652

[B6] Babatunde K. A. Subramanian B. Y. (2020). Role of Extracellular Vesicles in Cellular Cross Talk in Malaria. Front. Immunol. 11, 22. doi: 10.3389/fimmu.2020.00022 32082312 PMC7005784

[B7] Bautista-López N. L. Ndao M. Camargo V. (2017). Characterization and Diagnostic Application of Trypanosoma Cruzi Trypomastigote Excreted-Secreted Antigens Shed in Extracellular Vesicles Released From Infected Mammalian Cells. J. Clin. Microbiol. 55 (3), 744–585. doi: 10.1128/JCM.01649-16 PMC5328442 27974541

[B8] Bayer-SantosE.Aguilar-BonavidesC.RodriguesS. P.CorderoE. M.MarquesA. F.Varela-RamirezA.. (2013). Proteomic Analysis of Trypanosoma Cruzi Secretome: Characterization of Two Populations of Extracellular Vesicles and Soluble Proteins. J. Proteome Res. 12 (2), 883–975. doi: 10.1021/pr300947g 23214914

[B9] Beauvillain C. Ruiz S. Guiton R. Bout D. Dimier-Poisson I. (2007). A Vaccine Based on Exosomes Secreted by a Dendritic Cell Line Confers Protection Against *T. Gondii* Infection in Syngeneic and Allogeneic Mice. Microbes Infect. 9 (14–15), 1614–1622. doi: 10.1016/j.micinf.2007.07.002 17905628

[B10] Bozza P. T. Magalhães K. G. Weller Peter F. (2009). Leukocyte Lipid Bodies - Biogenesis and Functions in Inflammation. Biochim. Biophys. Acta 1791 (6), 540–551. doi: 10.1016/j.bbalip.2009.01.005 19416659 PMC2693476

[B11] Caler E. V. Morty R. E. Burleigh B. A. Andrews N. W. (2000). Dual Role of Signaling Pathways Leading to Ca2+ and Cyclic AMP Elevation in Host Cell Invasion by Trypanosoma Cruzi. Infect. Immun. 68 (12), 6602–6105. doi: 10.1128/IAI.68.12.6602-6610.2000 PMC97756 11083771

[B12] Campos F. M.F. Franklin B. S. Teixeira-carvalho A. Filho A. L.S. De Paula S. C. O. Fontes C. J.. (2010). Augmented Plasma Microparticles During Acute Plasmodium Vivax Infection. Malaria J. 9 (1), 3275. doi: 10.1186/1475-2875-9-327 PMC2998527 21080932

[B13] Cestari I. Ansa-Addo E. Deolindo P. Inal J. M. Ramirez M. I. (2012). *Trypanosoma cruzi* Immune Evasion Mediated by Host Cell-Derived Microvesicles. J. Immunol. 188 (4), 1942–1525. doi: 10.4049/jimmunol.1102053 22262654

[B14] Choudhuri S. Garg N. J. (2020). PARP1-CGAS-NF-KB Pathway of Proinflammatory Macrophage Activation by Extracellular Vesicles Released During Trypanosoma Cruzi Infection and Chagas Disease. PloS Pathog. 16 (4), 1–275. doi: 10.1371/journal.ppat.1008474 PMC7173744 32315358

[B15] Cronemberger-Andrade A. Aragão-França L. de Araujo C. F. Rocha V. J. Borges-Silva M. da C. Figueiras C. P.. (2014). Extracellular Vesicles From Leishmania-Infected Macrophages Confer an Anti-Infection Cytokine-Production Profile to Naïve Macrophages. PloS Neglect. Trop. Dis. 8 (9), 1–105. doi: 10.1371/journal.pntd.0003161 PMC4169249 25232947

[B16] Cronemberger-Andrade A. Xander P. Soares R. P. Pessoa N. L. Campos M. A. Cameron C.. (2020). *Trypanosoma cruzi*-Infected Human Macrophages Shed Proinflammatory Extracellular Vesicles That Enhance Host-Cell Invasion *via* Toll-Like Receptor 2. Front. Cell. Infect. Microbiol. 10, 99. doi: 10.3389/fcimb.2020.00099 32266161 PMC7098991

[B17] D’Avila H. Freire-de-Lima C. G. Roque N. R. Teixeira L. Barja-Fidalgo C. Silva A. R.. (2011). Host Cell Lipid Bodies Triggered by Trypanosoma Cruzi Infection and Enhanced by the Uptake of Apoptotic Cells Are Associated With Prostaglandin E2 Generation and Increased Parasite Growth. J. Infect. Dis. 204 (6), 951–615. doi: 10.1093/infdis/jir432 21849292

[B18] D’Avila H. Maya-monteiro C. M. Bozza P. T. (2008). Lipid Bodies in Innate Immune Response to Bacterial and Parasite Infections. Int. Immunopharmacol. 8 (10), 1308–1155. doi: 10.1016/j.intimp.2008.01.035 18687292

[B19] D’Avila H. Melo R. C. N. Parreira G. G. Werneck-Barroso E. Castro-Faria-Neto H. C. Bozza P. T. (2006). *Mycobacterium Bovis Bacillus Calmette-Guerin* Induces TLR2-Mediated Formation of Lipid Bodies: Intracellular Domains for Eicosanoid Synthesis *In Vivo* . J. Immunol. 176 (5), 3087–3097. doi: 10.4049/jimmunol.176.5.3087 16493068

[B20] De Pablos L. M. Lozano I. M. D. Jercic M. I. Quinzada M. Giménez M. J. Calabuig E.. (2016). The C-Terminal Region of *Trypanosoma cruzi* MASPs Is Antigenic and Secreted *via* Exovesicles. Sci. Rep. 6 (June), 1–12. doi: 10.1038/srep27293 27270330 PMC4897614

[B21] Dong G. Filho A. L. Olivier M. (2019). Modulation of Host-Pathogen Communication by Extracellular Vesicles (EVs) of the Protozoan Parasite *Leishmania* . Front. Cell. Infect. Microbiol. 9, 100. doi: 10.3389/fcimb.2019.00100 31032233 PMC6470181

[B22] Freire-De-Lima C. G. Nascimento D. O. Soares M. B. P. Bozza P. T. Castro-Faria-Neto H. C. De Mello F. G.. (2000). Uptake of Apoptotic Cells Drives the Growth of a Pathogenic Trypanosome in Macrophages. Nature, 403, 199–203. doi: 10.1038/35003208 10646605

[B23] Garcia-Silva M. R. Neves R. F. C. D. Cabrera-Cabrera F. Sanguinetti J. Medeiros L. C. Robello C.. (2014). Extracellular Vesicles Shed by Trypanosoma Cruzi Are Linked to Small RNA Pathways, Life Cycle Regulation, and Susceptibility to Infection of Mammalian Cells. Parasitol. Res. 113 (1), 285–304. doi: 10.1007/s00436-013-3655-1 24241124

[B24] Giri P. K. Schorey J. S. (2008). Exosomes Derived From M. Bovis BCG Infected Macrophages Activate Antigen-Specific CD4+ and CD8+ T Cells *In Vitro* and *In Vivo* . PloS One 3 (6), 1–105. doi: 10.1371/journal.pone.0002461 PMC2413420 18560543

[B25] Gonçalves M. F. Umezawa E. S. Katzin A. M. de Souza W. Alves M. J. M. Zingales B.. (1991). *Trypanosoma Cruzi*: Shedding of Surface Antigens as Membrane Vesicles. Exp. Parasitol. 72 (1), 43–535. doi: 10.1016/0014-4894(91)90119-H 1993464

[B26] Kalinski P. (2012). Regulation of Immune Responses by Prostaglandin E2. J. Immunol. 188 (1), 21–28. doi: 10.4049/jimmunol.1101029 22187483 PMC3249979

[B27] Kubata B. K. Duszenko M. Samuel Martin K. Urade Y. (2007). Molecular Basis for Prostaglandin Production in Hosts and Parasites. Trends Parasitol. 23 (7), 325–315. doi: 10.1016/j.pt.2007.05.005 17531535

[B28] Lidani K. C. F. Bavia L. Ambrosio A. R. de Messias-Reason I. J. (2017). The Complement System: A Prey of *Trypanosoma Cruzi* . Front. Microbiol. 8, 607. doi: 10.3389/fmicb.2017.00607 28473804 PMC5397499

[B29] Li Z. Thiel K. Thul P. J. Beller M. Kühnlein R. P. Welte M. A. (2012). Lipid Droplets Control the Maternal Histone Supply of Drosophila Embryos. Curr. Biol. 22 (22), 2104–2135. doi: 10.1016/j.cub.2012.09.018 23084995 PMC3513403

[B30] Lovo-Martins M. I. Malvezi A. D. Zanluqui N. G. Lucchetti B. F. C. Tatakihara V. L. H. Mörking P. A.. (2018). Extracellular Vesicles Shed By *Trypanosoma Cruzi* Potentiate Infection and Elicit Lipid Body Formation and PGE2 Production in Murine Macrophages. Front. Immunol. 9, 896 (Apr). doi: 10.3389/fimmu.2018.00896 29755471 PMC5934475

[B31] Lozano D. I. M. Pablos L. M. De Longhi S. A. Zago M. P. Schijman A. G. Osuna A. (2017). Immune Complexes in Chronic Chagas Disease Patients Are Formed by Exovesicles From *Trypanosoma Cruzi* Carrying the Conserved MASP N-Terminal Region. Sci. Rep. 7, 1–14. doi: 10.1038/srep44451 28294160 PMC5353755

[B32] Madeira R. P. Romera L. M. D. Buck P. De C. Mady C. Ianni B. M. Torrecilhas A. C. (2021). New Biomarker in Chagas Disease: Extracellular Vesicles Isolated From Peripheral Blood in Chronic Chagas Disease Patients Modulate the Human Immune Response. J. Immunol. Res. 11, 6650670. doi: 10.1155/2021/6650670 PMC7815414 33506056

[B33] Mantel P. Y. Marti M. (2014). The Role of Extracellular Vesicles in Plasmodium and Other Protozoan Parasites. Cell. Microbiol. 16 (3), 344–545. doi: 10.1111/cmi.12259 24406102 PMC3965572

[B34] Marcilla A. Martin-Jaular L. Trelis M. de Menezes-Neto A. Osuna A. Bernal D.. (2014). Extracellular Vesicles in Parasitic Diseases. J. Extracellular Vesicles 3 (1), 1–155. doi: 10.3402/jev.v3.25040 PMC4275648 25536932

[B35] Martins N. O. de Souza R. T. Cordero E. M. Maldonado D. C. Cortez C. Marini M. M.. (2015). Molecular Characterization of a Novel Family of Trypanosoma Cruzi Surface Membrane Proteins (TcSMP) Involved in Mammalian Host Cell Invasion. PloS Neglect. Trop. Dis. 9 (11), 1–28. doi: 10.1371/journal.pntd.0004216 PMC4643927 26565791

[B36] Melo R. C. N. Ávila H. D. Fabrino D. L. Almeida P. E. Bozza P. T. (2003). Macrophage Lipid Body Induction by Chagas Disease *In Vivo*: Putative Intracellular Domains for Eicosanoid Formation During Infection. Tissue Cell 35 (1), 59–67. doi: 10.1016/S0040-8166(02)00105-2 12589730

[B37] Moreira L. R. Serrano F. R. Osuna A. (2019). Extracellular Vesicles of Trypanosoma Cruzi Tissue-Culture Cell-Derived Trypomastigotes: Induction of Physiological Changes in Non-Parasitized Culture Cells. PloS Neglect. Trop. Dis. 13 (2), 1–265. doi: 10.1371/journal.pntd.0007163 PMC6383987 30789912

[B38] Murphy D. J. (2001). The Biogenesis and Functions of Lipid Bodies in Animals, Plants and Microorganisms. Progress Lipid Res 40 (5), 325–438. doi: 0.1016/S0163-7827(01)00013-3 10.1016/s0163-7827(01)00013-3 11470496

[B39] Murphy D. J. (2012). The Dynamic Roles of Intracellular Lipid Droplets: From Archaea to Mammals. Heterocyclic Commun. 249 (3), 541–585. doi: 10.1007/s00709-011-0329- 22002710

[B40] Neves R. F. C. Fernandes A. C. S. Meyer-Fernandes J. R. Souto-Padrón T. (2014). *Trypanosoma Cruzi*-Secreted Vesicles Have Acid and Alkaline Phosphatase Activities Capable of Increasing Parasite Adhesion and Infection. Parasitol. Res. 113 (8), 2961–2725. doi: 10.1007/s00436-014-3958-x 24906990

[B41] Nogueira P. M. de Menezes-Neto A. Borges V. M. Descoteaux A. Torrecilhas A. C. Xander P.. (2020). Immunomodulatory Properties of *Leishmania* Extracellular Vesicles During Host-Parasite Interaction: Differential Activation of TLRs and NF-κB Translocation by Dermotropic and Viscerotropic Species. Front. Cell. Infect. Microbiol. 10, 380. doi: 10.3389/fcimb.2020.00380 32850481 PMC7403210

[B42] Nogueira P. M. Ribeiro K. Silveira A. C. O. Campos J. H. Martins-Filho O. A. Bela S. R.. (2015). Vesicles From Different Trypanosoma Cruzi Strains Trigger Differential Innate and Chronic Immune Responses. J. Extracellular Vesicles 4 (1), 1–16. doi: 10.3402/jev.v4.28734 PMC4662668 26613751

[B43] Norris K. A. (1998). Stable Transfection of Trypanosoma Cruzi Epimastigotes With the Trypomastigote-Specific Complement Regulatory Protein CDNA Confers Complement Resistance. Infect. Immun. 66 (6), 2460–2465. doi: 10.1128/iai.66.6.2460-2465.1998 9596703 PMC108225

[B44] Norris K. A. Bradt B. Cooper N. R. So M. (1991). Characterization of a Trypanosoma Cruzi C3 Binding Protein With Functional and Genetic Similarities to the Human Complement Regulatory Protein, Decay-Accelerating Factor. J. Immunol. 147 (7), 2240–2247. 1717552

[B45] Oliveira L. Freire-de-lima G. Nosanchuk J. D. Casadevall A. Rodrigues M. L. Nimrichter L. (2010). Extracellular Vesicles From Cryptococcus Neoformans Modulate Macrophage Functions. Infect. Immun. 78 (4), 1601–1695. doi: 10.1128/IAI.01171-09 20145096 PMC2849392

[B46] Ostrowski M. Carmo N. B. Krumeich S. Fanget I. Raposo G. Savina A.. (2010). Rab27a and Rab27b Control Different Steps of the Exosome Secretion Pathway. Nat. Cell Biol. 12 (1), 19–30. doi: 10.1038/ncb2000 19966785

[B47] Palacios A. Gupta S. Rodriguez G.M. Prados-rosales R. (2021). Extracellular Vesicles in the Context of *Mycobacterium Tuberculosis* Infection. Mol. Immunol. 133 (Jan), 175–181. doi: 10.1016/j.molimm.2021.02.010 33743266 PMC9909588

[B48] Ramirez M. I. Deolindo P. de Messias-Reason I. J. Arigi E. A. Choi H. Almeida I. C.. (2017). Dynamic Flux of Microvesicles Modulate Parasite–Host Cell Interaction of Trypanosoma Cruzi in Eukaryotic Cells. Cell. Microbiol. 19 (4), 1–155. doi: 10.1111/cmi.12672 27665486

[B49] Rank A. Nieuwland R. Crispin A. Grützner S. Iberer M. Toth B.. (2011). Clearance of Platelet Microparticles *In Vivo* . Platelets 22 (2), 111–116. doi: 10.3109/09537104.2010.520373 21231854

[B50] Ribeiro K. S. Vasconcellos C. I. Soares R. P. Mendes M. T. Ellis C. C. Aguilera-Flores M.. (2018). Proteomic Analysis Reveals Different Composition of Extracellular Vesicles Released by Two Trypanosoma Cruzi Strains Associated With Their Distinct Interaction With Host Cells. J. Extracellular Vesicles 7, 213–228. doi: 10.1080/20013078.2018.1463779 PMC5912195 29696081

[B51] Scharfstein J. Schmitz V. Morandi V. Capella M. M. A. Lima A. P. C. A. Morrot A.. (2000). Host Cell Invasion by Trypanosoma Cruzi Is Potentiated by Activation of Bradykinin B2 Receptors. J. Exp. Med. 192 (9), 1289–1995. doi: 10.1084/jem.192.9.1289 11067878 PMC2193362

[B52] Silveira J.F. Da Abrahamsohn P. A. Colli W. (1979). Plasma Membrane Vesicles Isolated From Epimastigote Forms of Trypanosoma Cruzi. BBA - Biomembr. 550 (2), 222–325. doi: 10.1016/0005-2736(79)90209-8 365244

[B53] Silverman J. M. Clos J. Horakova E. Wang A. Y. Wiesgigl M. Kelly I.. (2010). Leishmania Exosomes Modulate Innate and Adaptive Immune Responses Through Effects on Monocytes and Dendritic Cells. J. Immunol. 185 (9), 5011–5022. doi: 10.4049/jimmunol.1000541 20881185

[B54] Tardieux I. Nathanson M. H. Andrews N. W. (1994). Role in Host Cell Invasion of Trypanosoma Cruzi-Induced Cytosolic-Free Ca2+ Transients. J. Exp. Med. 179 (3), 1017–1225. doi: 10.1084/jem.179.3.1017 8113670 PMC2191425

[B55] Théry C. Zitvogel L. Amigorena S. (2002). Exosomes: Composition, Biogenesis and Function. Nat. Rev. Immunol. 2 (8), 569–795. doi: 10.1038/nri855 12154376

[B56] Toledo D. A. M. Roque N. R. Teixeira L. Milán-Garcés E. A. Carneiro A. B. Almeida M. R.. (2016). Lipid Body Organelles Within the Parasite. Trypanosoma cruzi: A Role Intracellular Arachidonic Acid Metabolism. PloS One 11 (8), 1–22. doi: 10.1371/journal.pone.0160433 PMC4973985 27490663

[B57] Torrecilhas A. C. Schumacher R. I. Alves M. J. M. Colli W. (2012). Vesicles as Carriers of Virulence Factors in Parasitic Protozoan Diseases. Microbes Infect. 14 (15), 1465–1745. doi: 10.1016/j.micinf.2012.07.008 22892602

[B58] Torrecilhas A. C. Soares R. P. Schenkman S. Fernández-Prada C. Olivier M. (2020). Extracellular Vesicles in Trypanosomatids: Host Cell Communication.” Host Cell Communication. Front. Cell. Infect. Microbiol. 10, 602502. doi: 10.3389/fcimb.2020.602502 33381465 PMC7767885

[B59] Torrecilhas A. C. Tonelli R. R. Pavanelli W. R. da Silva J. S. Schumacher R. I. de Souza W.. (2009). *Trypanosoma Cruzi*: Parasite Shed Vesicles Increase Heart Parasitism and Generate an Intense Inflammatory Response. Microbes Infect. 11 (1), 29–39. doi: 10.1016/j.micinf.2008.10.003 19028594

[B60] Torró L. M. P. Moreira L. R. Osuna A. (2018). Extracellular Vesicles in Chagas Disease: A New Passenger for an Old Disease. Front. Microbiol. 9, 1190. doi: 10.3389/fmicb.2018.01190 29910793 PMC5992290

[B61] Van Niel G. Angelo G. D. Raposo G. (2018). Shedding Light on the Cell Biology of Extracellular Vesicles. Nat. Rev. Mol. Cell Biol. 19 (4), 1–16. doi: 10.1038/nrm.2017.125 29339798

[B62] Vargas G. Rocha J. D. Oliveira D. L. Albuquerque P. C. Frases S. Santos S. S.. (2015). Compositional and Immunobiological Analyses of Extracellular Vesicles Released by Candida Albicans. Cell. Microbiol. 17 (3), 389–407. doi: 10.1111/cmi.12374 25287304

[B63] Walther T. C. Chung J. Farese R. V. Jr. (2017). Lipid Droplet Biogenesis. Annu. Rev. Cell Dev. Biol. 33 (Oct), 491–5100. doi: 10.1146/2Fannurev-cellbio-100616-060608 28793795 PMC6986389

[B64] Witwer K. W. Théry C. (2019). Extracellular Vesicles or Exosomes? On Primacy, Precision, and Popularity Influencing a Choice of Nomenclature. J. Extracellular Vesicles 8 (1), 1–7. doi: 10.1080/20013078.2019.1648167 PMC6711079 31489144

[B65] Yoshida N. Cortez M. (2008). *Trypanosoma Cruzi*: Parasite and Host Cell Signaling During the Invasion Process. Subcellular Biochem. 47, 82–91. doi: 10.1007/978-0-387-78267-6_6 18512343

